# A decade of viral mutations and associated drug resistance in a population of HIV-1^+^ Puerto Ricans: 2002–2011

**DOI:** 10.1371/journal.pone.0177452

**Published:** 2017-05-11

**Authors:** Lycely del C. Sepúlveda-Torres, Lavanya Rishishwar, Maria Luisa Rogers, Eddy Ríos-Olivares, Nawal Boukli, I. King Jordan, Luis A. Cubano

**Affiliations:** 1 Department of Microbiology and Immunology, Universidad Central del Caribe, Bayamón, Puerto Rico, United States of America; 2 School of Biological Sciences, Georgia Institute of Technology, Atlanta, Georgia, United States of America; 3 PanAmerican Bioinformatics Institute, Cali, Valle del Cauca, Colombia; 4 Applied Bioinformatics Laboratory, Atlanta, Georgia, United States of America; 5 Natural Sciences Department, Sacred Heart University, San Juan, Puerto Rico, United States of America; National Institutes of Health, UNITED STATES

## Abstract

Puerto Rico has one of the highest rates of HIV/AIDS seen for any US state or territory, and antiretroviral therapy has been a mainstay of efforts to mitigate the HIV/AIDS public health burden on the island. We studied the evolutionary dynamics of HIV-1 mutation and antiretroviral drug resistance in Puerto Rico by monitoring the population frequency of resistance-associated mutations from 2002 to 2011. Whole blood samples from 4,475 patients were analyzed using the TRUGENE HIV-1 Genotyping Kit and OpenGene DNA Sequencing System in the Immunoretrovirus Research Laboratory at Universidad Central del Caribe. Results show that 64.0% of female and 62.9% of male patients had HIV-1 mutations that confer resistance to at least one antiretroviral medication. L63P and M184V were the dominant mutations observed for the protease (PRO) and reverse transcriptase (RT) encoding genes, respectively. Specific resistance mutations, along with their associated drug resistance profiles, can be seen to form temporal clusters that reveal a steadily changing landscape of resistance trends over time. Both women and men showed resistance mutations for an average of 4.8 drugs over the 10-year period, further underscoring the strong selective pressure exerted by antiretrovirals along with the rapid adaptive response of HIV. Nevertheless, both female and male patients showed a precipitous decrease for overall drug resistance, and for PRO mutations in particular, over the entire course of the study, with the most rapid decrease in frequency seen after 2006. The reduced HIV-1 mutation and drug resistance trends that we observed are consistent with previous reports from multi-year studies conducted around the world. Reduced resistance can be attributed to the use of more efficacious antiretroviral drug therapy, including the introduction of multi-drug combination therapies, which limited the ability of the virus to mount rapid adaptive responses to antiretroviral selection pressure.

## Introduction

Since the beginning of the HIV/AIDS epidemic through April 2016, a total of 47,722 new cases have been confirmed in Puerto Rico, 10,927 of which were diagnosed in the 10-year period from 2002 to 2011 [1]. Official statistics compiled by the US Centers for Disease Control and Prevention (CDC) underscore the extremely high public health burden imposed by HIV/AIDS in Puerto Rico. The estimated HIV diagnosis rate for adolescents and adults was 29.5 per 100,000 inhabitants in 2002 and 24.0 in 2011, ranking Puerto Rico as the 5^th^ and 7^th^ highest out of 56 US states and territories for those years [2, 3]. Almost 75% of the epidemic is concentrated in the metropolitan area of San Juan/Carolina/Caguas, which is the most populated region in Puerto Rico. San Juan was ranked as the 9^th^ highest metropolitan area in the US for new HIV infections in 2002, with an HIV diagnosis rate of 35.5, and 13^th^ in the nation in 2011, with a diagnosis rate of 26.6 [2, 3].

The introduction of highly active antiretroviral therapy (HAART) into clinical practice in the late 1990s, coupled with early diagnosis and treatment of HIV infection, led to dramatic reductions in HIV morbidity and mortality, better health outcomes for infected individuals and reduced transmission of HIV to partners [4–6]. From the introduction of antiretroviral therapy until 2010, the annual number of deaths in the US due to AIDS dropped by 85%, including a 14% decrease between 2009 and 2010 alone [7, 8]. Nevertheless, Hispanics/Latinos living in Puerto Rico and on the mainland remain at significantly greater risk for delayed diagnosis of HIV and AIDS compared to non-Hispanic whites, which greatly compromises their access to effective care and treatment [9, 10]. Accordingly, individuals born in Puerto Rico have twice the risk of HIV-related deaths than US-born patients and are even at a higher risk of mortality compared to other Hispanic populations [11, 12]. Vital statistics reported for the testing period covered in this study place AIDS as the 13^th^ leading cause of death for Puerto Ricans living on the island and 17^th^ for Hispanics living in the mainland [13, 14].

Guidelines for the use of antiretroviral agents in HIV-1 infected adolescents and adults call for personalized treatments, whereby specific medications are selected based on the predicted resistance profile for the viral mutation(s) present in the patient, along with other considerations related to comorbidities, dosing frequency and burden, adverse drug interactions, and toxic effects [15, 16]. Current HIV/AIDS treatment protocols call for the essential viral genes that encode for the protease (PRO) and reverse transcriptase (RT) proteins to be sequenced and analyzed in order to detect mutations that are known to confer antiretroviral drug resistance, thereby allowing a suitable combination of antiretroviral drugs to be prescribed. The Universidad Central del Caribe (UCC) provided HIV genome sequencing and drug resistance analysis services for this purpose in Puerto Rico from 2000 to 2011, yielding a unique and valuable data source for research on the evolutionary dynamics of HIV-1 drug resistance on the island. While the UCC HIV-1 antiretroviral drug resistance data for individual years has been published elsewhere [17–19], prior to this time we had yet to attempt a multi-year analysis of resistance trends in Puerto Rico. For the current study, we took a data analysis approach to examine the changing prevalence of HIV-1 resistance-associated mutations in Puerto Rico over the 10-year period from 2002 to 2011. The previously published differences in viral mutations and drug resistance levels between men and women for individual years did not remain significant when the cumulative 10-year dataset was analyzed. Nevertheless, our statistical analysis of HIV-1 resistance mutations over this time frame revealed a number of clear dynamic trends, including a sharp reduction in the frequency of drug resistance mutations over the last 5 years of the 10-year span.

## Materials and methods

### HIV-1 genotyping

**Fig 1:**
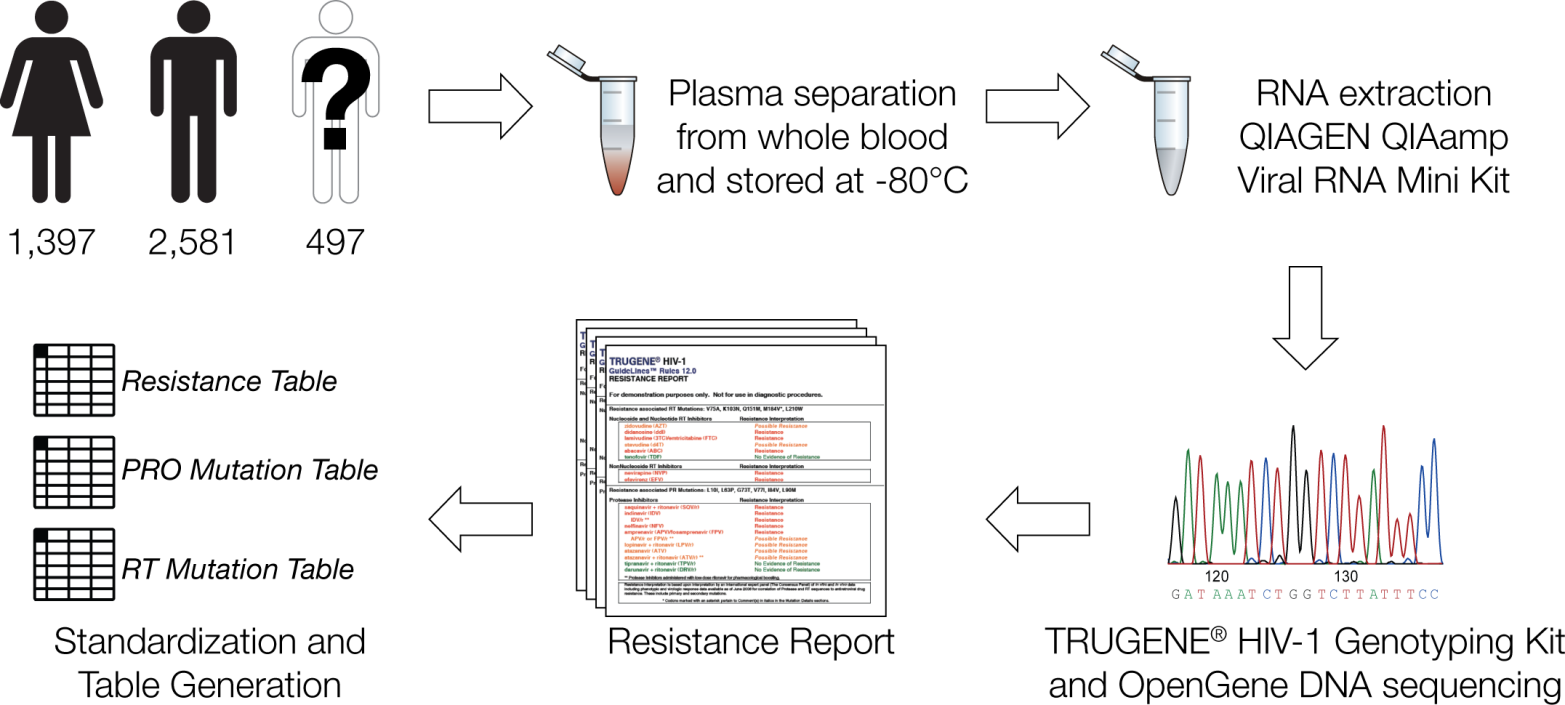
Cohort sample analysis and HIV-1 genotyping workflow used for the study.

HIV-1 genotypes were obtained from 1,397 female, 2,581 male and 497 anonymous HIV-1 infected patients who were referred by their primary care physicians to the Immunoretrovirus Research Laboratory at Universidad Central del Caribe from 2002 to 2011. The samples were analyzed using the protocol illustrated in Fig 1 after the IRB Director at Universidad Central del Caribe deemed the research exempt from the human subjects in research regulations. Whole blood was collected in tubes containing ethylenediaminetetraacetic acid (EDTA) as anticoagulant, followed by plasma separation and storage at -80°C. HIV-1 viral RNA was extracted using the QIAGEN QIAamp Viral RNA Mini Kit (QIAGEN, Valencia CA) as previously described [19]. RNA samples were analyzed with the TRUGENE HIV-1 Genotyping Kit and OpenGene DNA Sequencing System equipped with a proprietary interpretative algorithm that infers resistance to specific antiretroviral drugs from the observed mutations based on prior knowledge (Siemens Healthcare Diagnostics, Tarrytown, NY).

### Quantitative analysis of HIV-1 mutations and drug resistance trends

**Fig 2:**
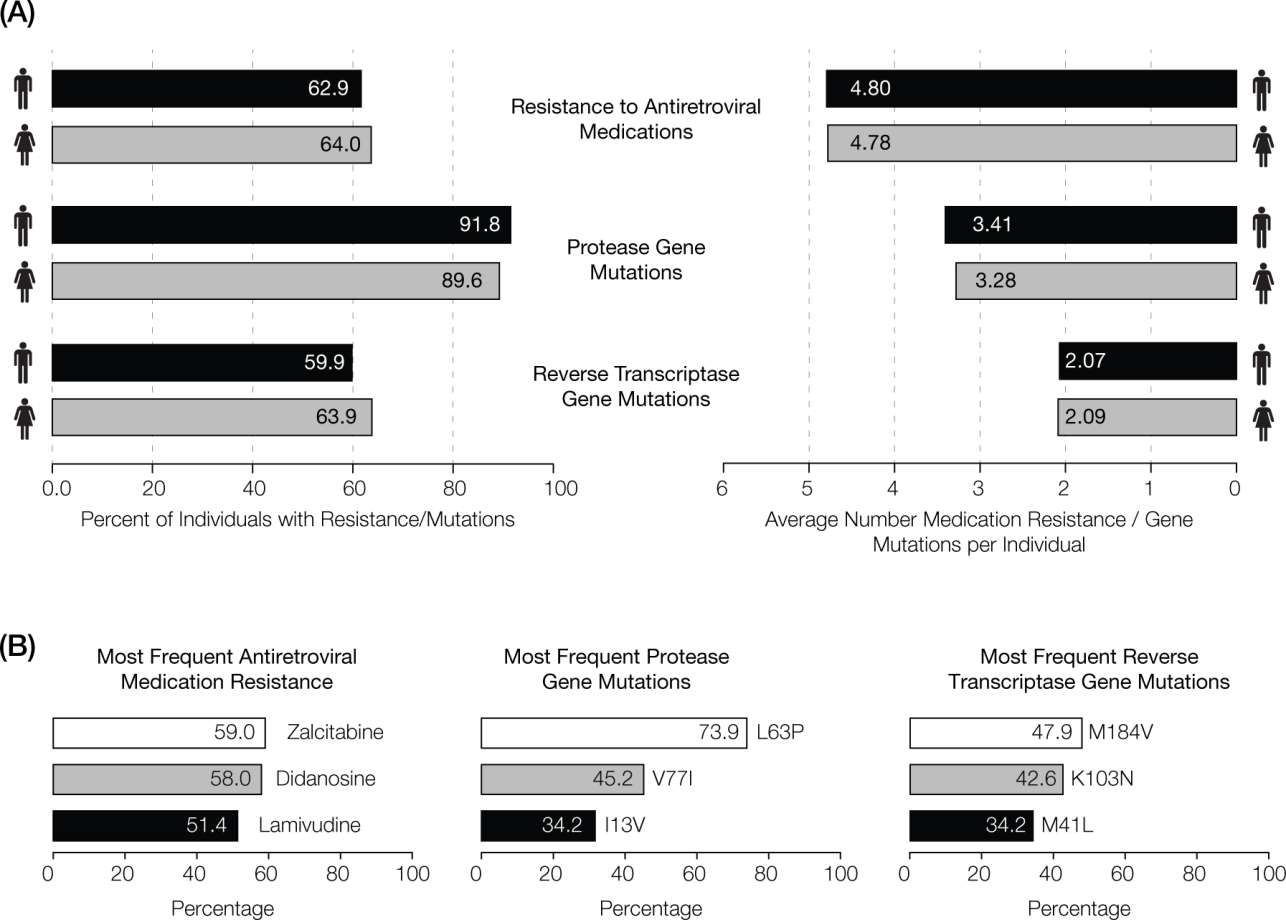
Observed levels of antiretroviral resistance-associated mutations in Puerto Rico from 2002 to 2011. **(A)** Drug resistance and mutation frequencies recorded by gender. **(B)** Percentages of individuals seen for the most commonly observed antiretroviral drug resistance and the most frequent mutations in the protease (PRO) and reverse transcriptase (RT) genes.

Information on HIV-1 drug resistance profiles and mutations was taken from de-identified patient TRUGENE HIV-1 Genotyping Kit resistance reports and used to create three databases for (1) drug resistance profiles, (2) protease (PRO) gene mutations, and (3) reverse transcriptase (RT) gene mutations (Fig 1). Patient-isolated viral nucleic acids were screened for resistance to 26 HIV drugs based on 104 PRO mutations and 62 RT mutations. Each database contained individual patient binary presence/absence scores for all the drug resistance profiles and mutations screened during the 2002 to 2011 period. Gender was the only demographic information provided by patients and was included as metadata in the databases. Summary statistics on the overall prevalence of drug resistance and HIV-1 mutations were calculated from the three patient databases to generate the data shown in Fig 2. The statistical significance of gender differences between drug resistance and mutation trends was tested using the Fisher’s exact test. The resulting *P*-values were adjusted via the false discovery rate, calculated using the Benjamini-Hochberg procedure, as a correction for multiple statistical tests.

Additional statistical analyses of the databases were done to compare the dynamics of the drug resistance profiles and HIV-1 mutations for the PRO and RT genes over time. For each drug, the annual drug resistance fraction (*d*_*res*_) was calculated as the number of individuals who tested positive for drug resistance for a given year ($n_{res}^{i}$) normalized by the total number of individuals screened that year ($n_{tot}^{i}$):

$$d_{res}=n_{res}^{i}/n_{tot}^{i}$$ (1)

In order to compare the year-to-year dynamics of resistance between different drugs, an additional calculation was performed to yield normalized drug resistance fractions ($\hat{d_{res}}$). For each drug, the annual *d*_*res*_ values were normalized by the maximum value (max(*d*_*res*_)) over all years for that drug.

$$\hat{d_{res}}=d_{res}/max(d_{res})$$ (2)

For each patient, the annual patient drug resistance fraction (*p*_*res*_) was computed as the number of drugs for which the patient was found to be resistant in a given year ($n_{res}^{d}$) normalized by the total number of drugs screened for that patient ($n_{tot}^{d}$).

$$p_{res}=n_{res}^{d}/n_{tot}^{d}$$ (3)

In order to compare the year-to-year dynamics of patient drug resistance, the average values for annual patient drug resistance fractions were calculated. This was done for all patients as well as separately for males and females.

$$\bar{p_{res}}=\sum p_{res}/n_{tot}^{i}$$ (4)

The annual dynamics of HIV-1 resistance mutations in PRO and RT were calculated identically as described above for the normalized drug resistance fractions (Eqs 1 and 2). In the case of the PRO and RT mutations, the calculations were made based on the number of individuals who tested positive for a given mutation in a given year ($n_{mut}^{i}$) normalized by the total number of individuals tested that year ($n_{tot}^{i}$).

$${PRO}_{mut}=n_{mut}^{i}/n_{tot}^{i}$$ (5)

$$\hat{{PRO}_{mut}}={PRO}_{mut}/max({PRO}_{mut})$$ (6)

$${RT}_{mut}=n_{mut}^{i}/n_{tot}^{i}$$ (7)

$$\hat{{RT}_{mut}}={RT}_{mut}/max({RT}_{mut})$$ (8)

Only the most common 49 PRO and 51 RT mutations were selected for subsequent analysis, as the remaining low frequency mutations cannot be accurately analyzed with respect to any dynamic trends over time.

## Results

### Overview of HIV-1 drug resistance and mutation trends in Puerto Rico from 2002 to 2011

HIV-1 genotypes and associated drug resistance profiles were characterized for 4,475 patients over a 10-year period in Puerto Rico using the approach shown in Fig 1, as described in the Materials and Methods section. S1 Table contains a complete list of the counts of individuals showing protease (PRO) and reverse transcriptase (RT) mutations, along with their associated drug resistance counts, broken down by gender and year. The most commonly observed mutations and their associated antiretroviral resistant profiles are summarized in Fig 2. HIV-1 virus strains harboring at least one antiretroviral drug resistance-associated mutation were found in 64.0% of women and 62.9% of men; both women and men showed resistance to an average of ~4.8 drugs over the 10-year period (Fig 2). 89.6% of women and 91.8% of men were infected with strains containing at least one PRO gene mutation, with corresponding resistance to an average of 3.3 PRO inhibitor drugs for women and 3.4 for men. 63.9% of women had at least one RT gene mutation compared to 59.9% of men, with corresponding resistance to an average of 2.1 RT medications for both. We checked for gender differences in drug resistance and mutations across each individual year using the Fisher’s exact test. After correcting for multiple statistical tests using the false discovery rate, no drugs or mutations showed statistically significant gender differences for any single year or for the entire 10-year period.

### HIV-1 antiretroviral drug resistance trends

**Fig 3:**
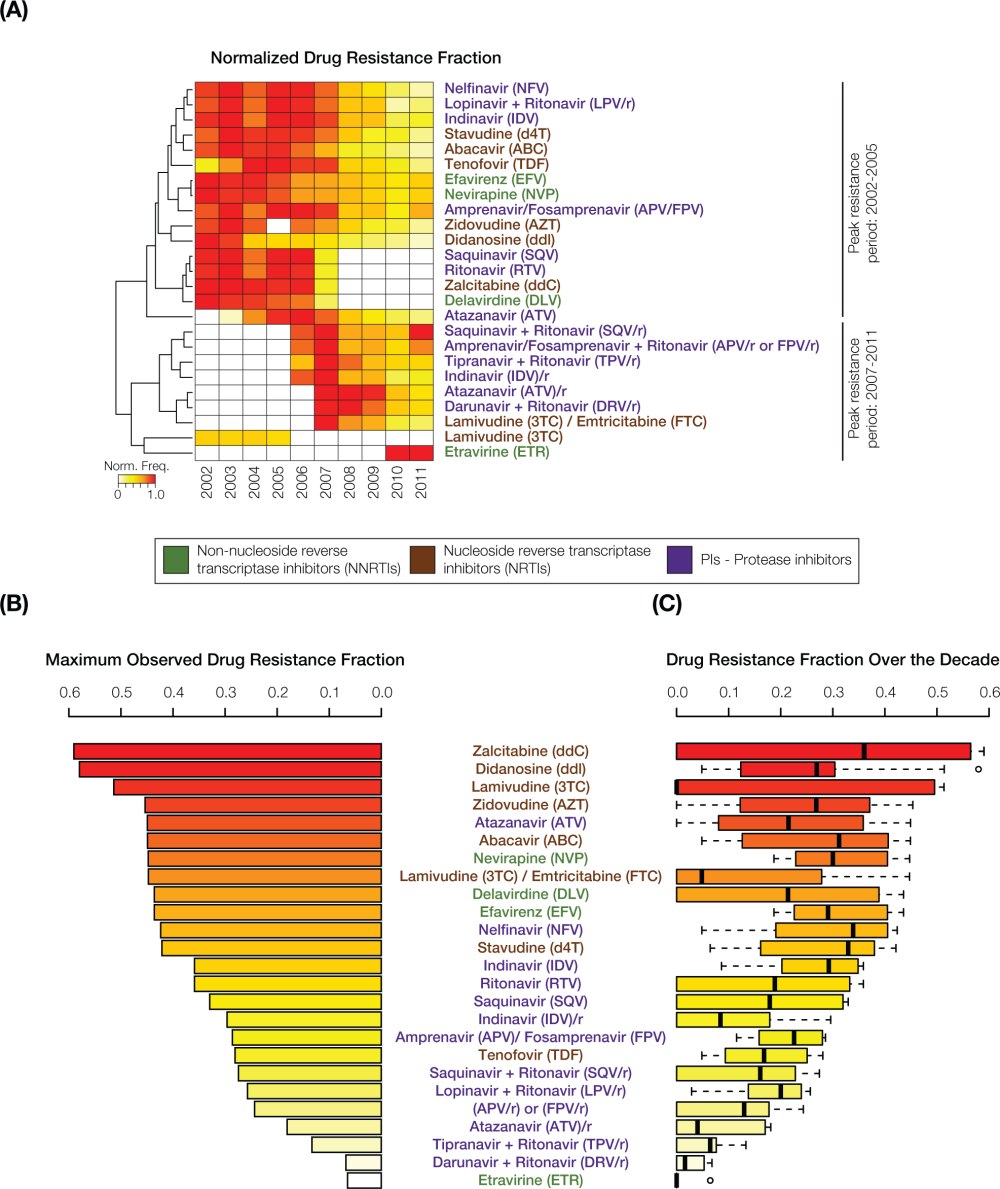
Trends in HIV-1 drug resistance levels in Puerto Rico from 2002 to 2011. The names of the antiretroviral drugs are color-coded with respect to their class of action as shown in the key in the middle of the figure. **(A)** Normalized drug resistance frequencies, color-coded as shown in the key, are shown for the drugs analyzed here over the 2002–2011 period. White boxes indicate an absence of resistance mutations for a drug in any given year. Normalized drug resistance fraction profiles are grouped using hierarchical clustering, revealing two clusters with distinct peak resistance periods. **(B)** The maximum observed resistance fractions are shown for all drugs alongside **(C)** box-plot distributions of the maximum observed resistance fractions.

HIV-1 resistance levels to antiretroviral drugs were inferred from PRO and RT gene mutations characterized using the TRUGENE HIV-1 Genotyping Kit as described in the Materials and Methods section. Hierarchical clustering and presentation of normalized drug resistance levels revealed two distinct time periods that overlap slightly around 2007, each of which is characterized by peak resistance levels to different sets of drugs (Fig 3). We did not observe any clear relationship between the class of action of the antiretroviral drugs and the two peak time periods. The highest antiretroviral drug resistance levels over the 10-year period were observed for Zalcitabine (59.0%), Didanosine (58.0%) and Lamivudine (51.4%) (Figs 2 and 3), whereas Tipranavir + ritonavir (TPV/r), Darunavir + ritonavir (DRV/r) and Etravirine (ETR) showed the lowest levels of resistance.

### Protease (PRO) resistance-associated mutation trends

**Fig 4:**
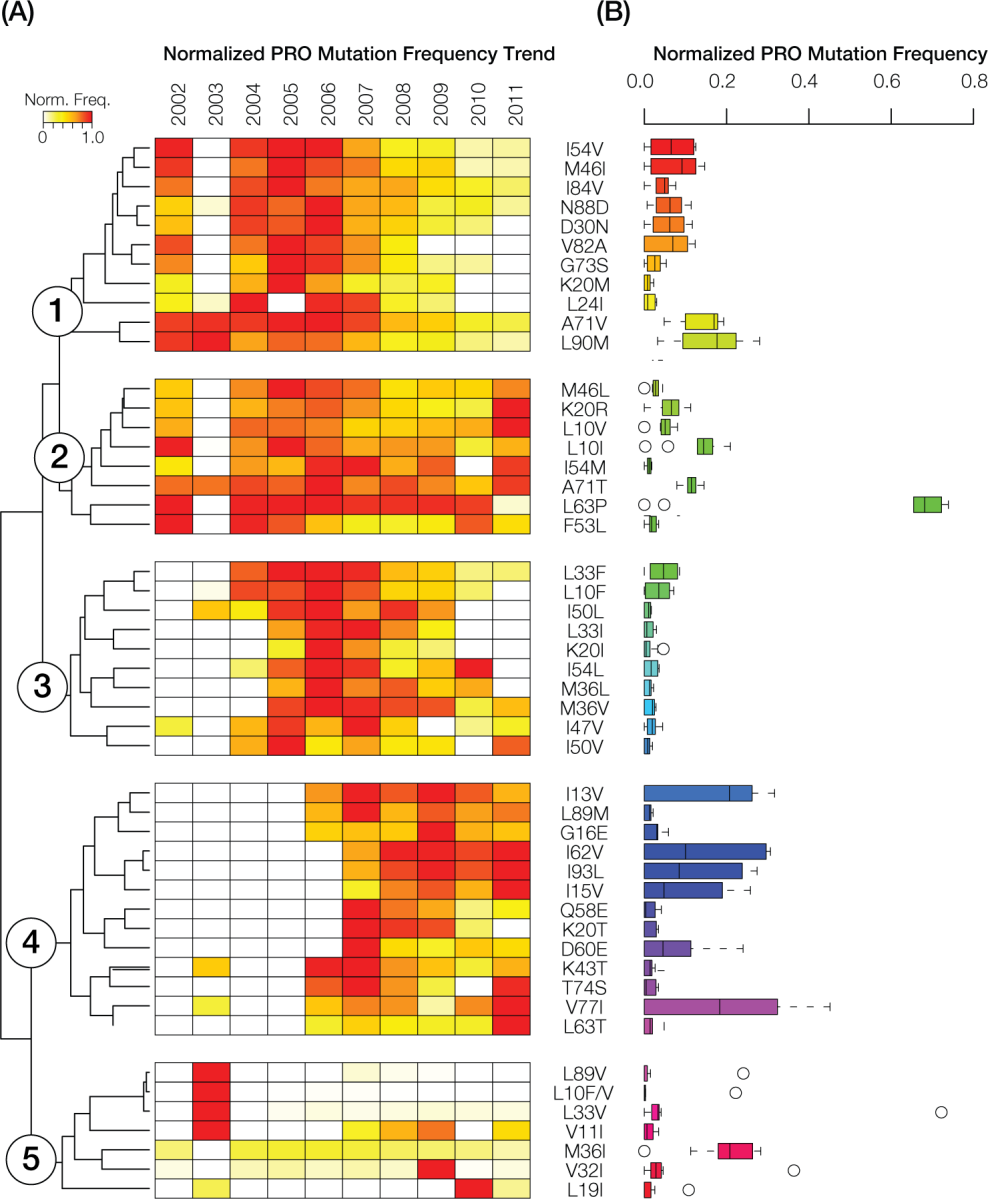
Trends in HIV-1 protease mutations in Puerto Rico from 2002 to 2011. **(A)** Normalized protease (PRO) mutation frequencies, color-coded as shown in the key, are shown for the 2002–2011 period. White boxes indicate an absence of PRO resistance mutations in any given year. PRO mutation frequency profiles are grouped using hierarchical clustering, revealing five distinct clusters. Mutation names are shown adjacent to the clustering plot. **(B)** Box-plot distributions of the normalized PRO mutation frequencies. Box-plots are color-coded using a rainbow scheme to visually distinguish the five PRO mutation frequency clusters.

We performed hierarchical clustering of normalized PRO mutation frequencies for the 49 most common mutations in order to visualize their evolutionary dynamics. PRO mutation trends were found to be highly dynamic over the 10-year time period and can be resolved into five distinct mutation groups based on the hierarchical clustering (Fig 4). Class 1 had peak frequencies prior to 2007 followed by a steady and gradual decrease through the end of the 10-year period. Class 2 shows a similar trend as seen for class 1 with the exception of the re-emergence of 2 mutations in 2010 and 6 mutations in 2011. Class 3 peak frequencies were observed around 2006–2007, whereas class 4 mutations were virtually absent prior to that time and peaked afterwards. Class 4 mutations are also characterized by their relatively high average frequency over this second time period. Class 5 shows the most internal divergence with 4 mutations that peaked markedly in one year, whereas the remaining mutations are observed at uniformly low to moderate frequencies across all years, including the M36I mutation that stands out by virtue of its sustained moderate frequency level. The 3 most prevalent HIV-1 resistance-associated mutations for PRO were L63P (73.9%), V77I (45.2%) and I13V (34.2%), whereas I50L, I50V and K20I were the least abundant mutations recorded during the 10-year period (Figs 2 and 4).

### Reverse transcriptase (RT) resistance-associated mutation trends

**Fig 5:**
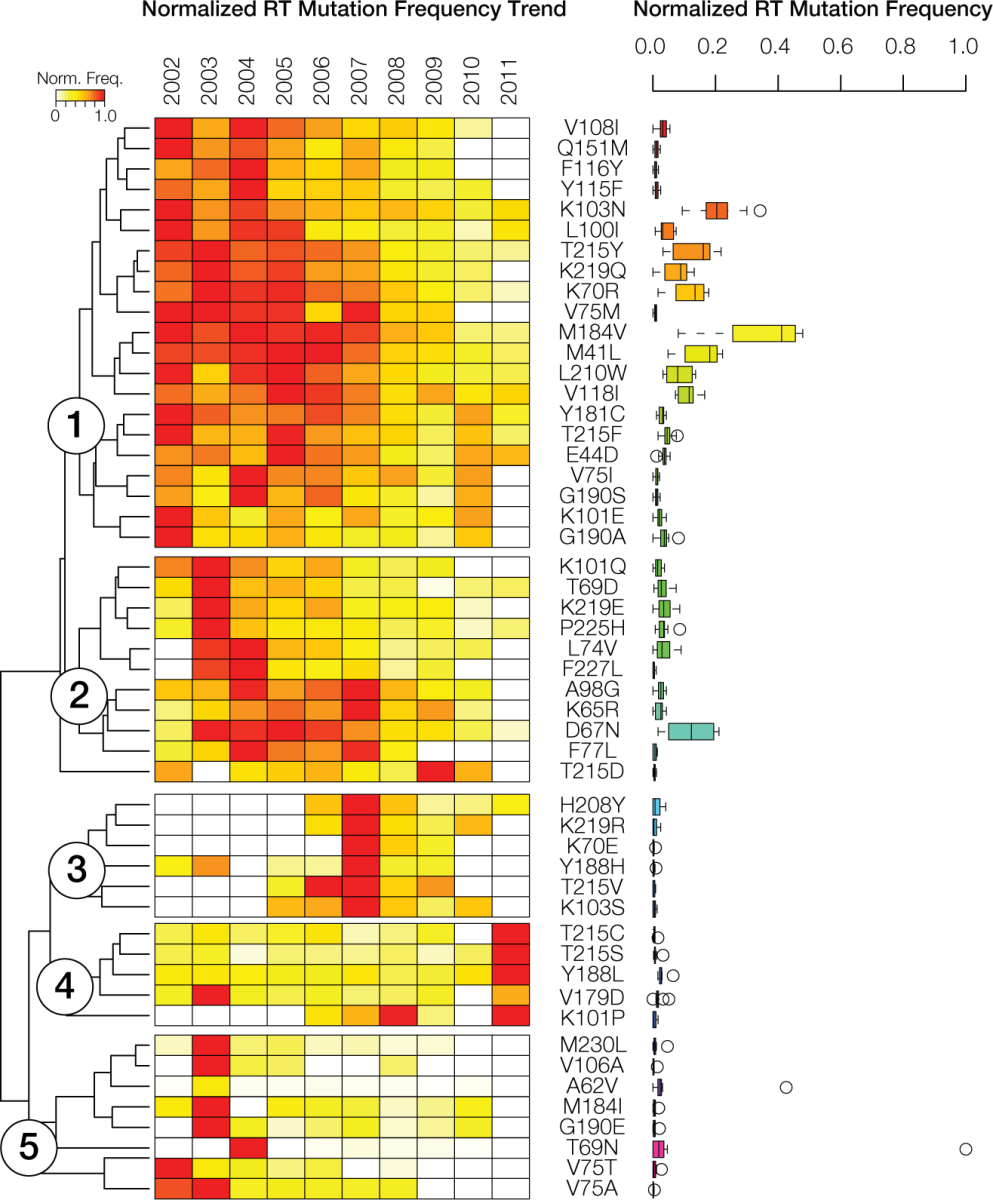
Trends in HIV-1 reverse transcriptase mutations in Puerto Rico from 2002 to 2011. **(A)** Normalized reverse transcriptase (RT) mutation frequencies, color-coded as shown in the key, are shown for the 2002–2011 period. White boxes indicate an absence of RT resistance mutations in any given year. RT mutation frequency profiles are grouped using hierarchical clustering, revealing five distinct clusters. Mutation names are shown adjacent to the clustering plot. **(B)** Box-plot distributions of the normalized RT mutation frequencies. Box-plots are color-coded using a rainbow scheme to visually distinguish the five RT mutation frequency clusters.

We also performed hierarchical clustering of normalized RT mutation frequencies for the 51 most common mutations in order to visualize their evolutionary dynamics. RT mutation trends are similarly dynamic, and even more variable across years, compared to what was observed for PRO mutations (Fig 5). RT mutations can also be grouped into five classes using hierarchical clustering; although, the dynamics among these are slightly different compared to those observed for PRO. Overall, there is a higher frequency of RT mutations early in the time period followed by a subsequent decline in frequency. This pattern is exemplified by Class 1 and Class 2, both of which were highly frequent until 2007, followed by a decline in frequencies thereafter. These two classes also contain the mutations that are seen in the highest frequency across the entire time period. The remaining three classes all show marked mutation peaks in a single year coupled with relatively low frequency levels over the rest of the period. Class 4 mutations peak in 2011 and show moderate frequencies for the rest of the period. Class 5 has exclusive mutation frequency peaks in the first two years of the study (2002–2003), followed by low frequencies over the rest of the period. The RT mutations with the highest average frequencies were M184V (47.9%), K103N (42.6%) and M41L (34.2%) (Figs 2 and 5); the three least common RT mutations over the 10-year period were K103T, Y188C and P236L (S1 Table).

## Discussion

### Functional implications of the most common PRO and RT mutations

Previous research shows that the most common PRO mutation observed in the Puerto Rican sample–L63P –which is estimated to occur in half of drug-naïve patients, provides a strong adaptive benefit to HIV viruses replicating under drug pressure [20]. Indeed, the same PRO position 63 shows an array of amino acid substitutions that alter the site of the enzyme that is bound by antiretroviral drugs, thereby decreasing the drugs’ affinity and efficacy [21]. The second most frequent PRO mutation that we observed in Puerto Rico–V77I –is considered to be one of the most prominent non-active site mutations implicated in antiretroviral drug resistance. The V77I mutation facilitates increased drug resistance by providing conformational flexibility to the enzyme [22]. The third most abundant PRO mutation that we observed–I13V –is associated with fast virologic failure and decreased drug affinity [23]. Finally, M36I, a PRO mutation whose frequency remained relatively constant over the period of study, in both Puerto Rico and throughout Latin America, influences the flexibility of the protease and its complexed substrate, thereby mediating interactions linked to increased drug resistance [24, 25].

The most prevalent RT mutations observed in Puerto Rico during the 10-year period of this study were also the dominant mutations identified in a meta-analysis of HIV drug resistance trends in Latin America and the Caribbean between 2000 and 2015 [25]; it should be noted that the dataset used for the meta-analysis is distinct from the UCC mutation dataset that we analyzed here. The predominant RT mutation observed in this study–M184V –is widely considered to be the most critical mutation with respect to resistance to multiple nucleoside and nucleotide analog RT inhibitors (NRTIs) [26, 27]. The presence of K103N, the second most common RT mutation that we observed, diminishes the efficacy of non-nucleoside analog RT inhibitors (NNRTIs) and, unlike other NNRTI-resistance mutations, persists for a prolonged period of time after the initial viral infection [27]. The third most common RT mutation that we observed–M41L –also confers resistance to NRTIs by helping to restore DNA polymerase activity to RT proteins that have been disabled by other mutations [28].

### Potential sources of bias in the observed drug resistance trends

Our study is dependent upon the results generated by the TRUGENE HIV-1 Genotyping Kit and OpenGene DNA sequencing system, which relies on a proprietary interpretative algorithm to generate the output that was used for the statistical analyses that we report. It is important to note that the accuracy of this system has been independently confirmed several times [29–31], including as part of a rigorous FDA approval process [32]. Nevertheless, the propriety software used by the TRUGENE kit was updated 6 times between 2002 and 2011, primarily in an effort to reflect the most current knowledge of HIV-1 gene mutation to drug resistance associations. These updates, together with any changes made in clinical practice, could introduce technical artifacts that may appear as dynamic trends based on the statistical analyses that we used to analyze HIV mutations and their associated drug resistance frequencies over time.

In an effort to evaluate this possible bias, we compared the results obtained from our statistical analyses with data published by the International Antiretroviral Society (IAS) for 2001 to 2012, which associate HIV mutations with resistance to specific antiretroviral drugs [33–43]. According to the IAS tables published from 2001 to 2004, the most frequent mutation that we observed for the RT gene–M184V –confers resistance to Zalcitabine, which also appears as the most frequent drug resistance in our analysis (Fig 3). The IAS data also associates M184V with the antiretrovirals Didanosine and Lamivudine, which were the second and third most frequent medications observed for our samples. These consistencies provide an additional line of support for the TRUGENE HIV-1 Genotyping Kit that we used. On the other hand, Zalcitabine was removed from the market in 2006, which explains why it disappears completely from our data after 2007, even though it could be inferred from Fig 3 that no resistance was observed for this medication in the last years of the study. Thus, missing mutation and/or resistance data from our study should be interpreted with some caution.

The same IAS data reveal several other potentially confounding factors regarding HIV-1 mutation-to-drug resistance associations that we studied. For example, the antiretrovirals Indinavir, Saquinavir and Ritonavir are listed individually from 2001 to 2005, but are shown in combination thereafter: Indinavir/ritonavir and Saquinavir/ritonavir. This change was made in response to the introduction of drug combinations to the market, which reduced pill burden while maintaining safe and effective viral control [44]. Tipranavir is absent from our analysis prior to 2006 because it was approved in 2005. Finally, Delavirdine was no longer recommended as part of an initial therapy regime from 2006, owing to its inconvenient dose schedule and lower efficacy when compared with other medications [45]; this explains its sudden decline in frequency observed for the last years of our study.

## Conclusions

**Fig 6:**
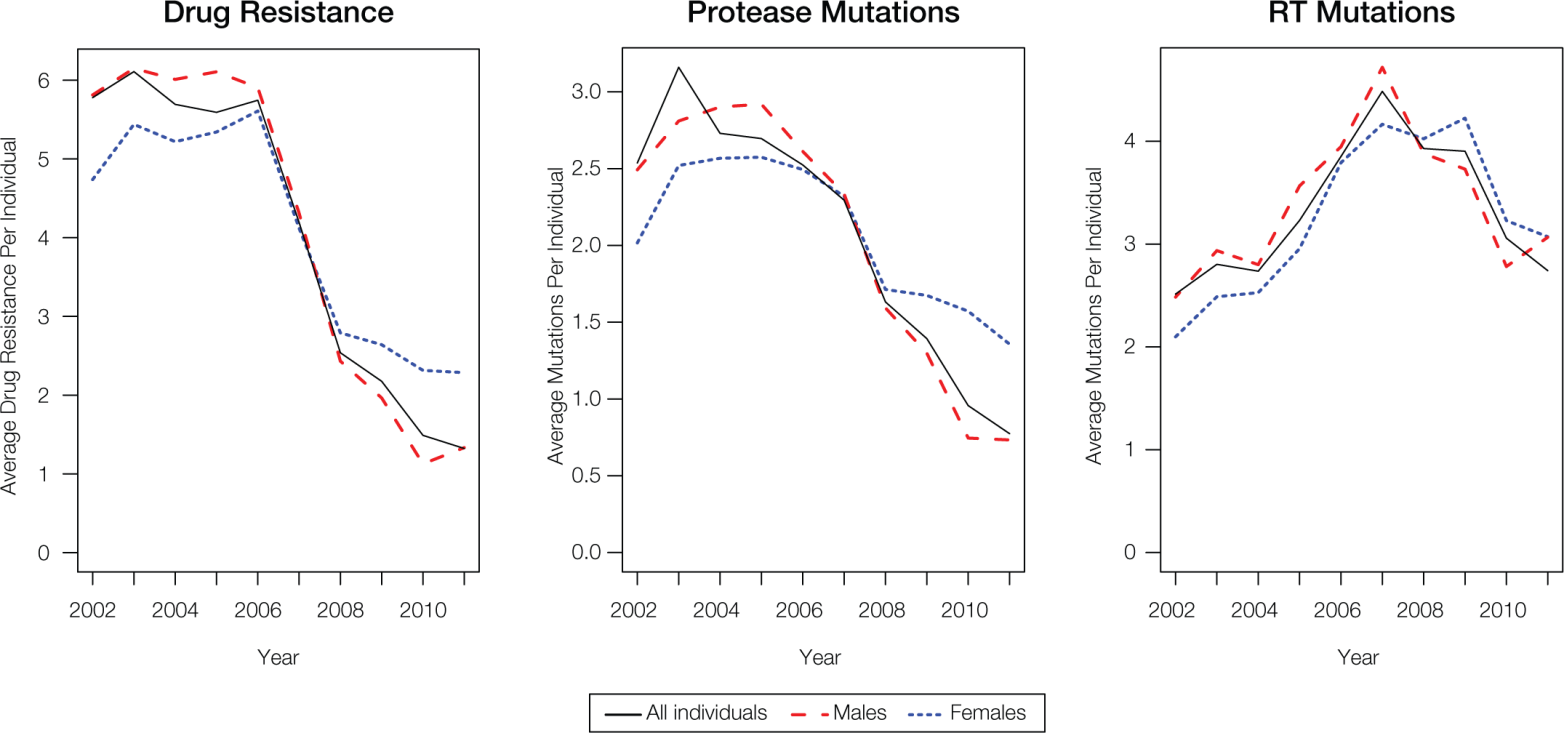
Average number of drug resistance or mutations per individual during the 2002 to 2011 period. Values were computed for all individuals (black-solid), females (blue-dotted), and males (red-dashed).

HIV-1 mutation and drug resistance trends in Puerto Rico were found to be highly dynamic over the 10-year period of the study. For both women and men, a marked decrease was seen for overall drug resistance and for protease mutations in particular, with the sharpest decline occurring after 2006 (Fig 6). The reduced HIV-1 mutation and drug resistance trends that we observed are consistent with previous reports from multi-year cohort studies conducted around the world for the same time period [46–49]. Our results are also consistent with population genetic studies, which contend that the transition from early HIV drug therapies to the newer, more efficacious drug treatment regimes, including multi-drug cocktails, limited the ability of the virus to mutate and evolve drug resistance. Possible underlying reasons for the decline in HIV drug resistance include reduced viral load, the low probability of simultaneously evolving combinations of mutations to evade multiple drugs, more effective treatment guidelines and better treatment adherence [50, 51].

Interestingly, the prospect of selecting for resistant strains of HIV, along with elevated transmission rates of resistant virus in newly infected patients, were foreseen consequences of the broad implementation of HAART. On the other hand, our analyses concur with other studies that report declines in drug resistance following expansion of treatment, pointing to the ability of antiretroviral therapy to halt viral evolution [52, 53]. It has even been shown that the emergence of drug resistance can be virtually stopped when newer and more potent therapies are coupled with close patient monitoring [54]. These findings, considered together with the results of our own study reported here, underscore the critical importance of prospective cohort monitoring for combating the HIV/AIDS epidemic in Puerto Rico. Active surveillance of HIV-1 mutation and associated drug resistance trends are critical components that are needed to ensure the successful treatment of HIV infected patients on the island.

## Supporting information

S1 Table HIV-1 mutations and drug resistance in Puerto Rico from 2002 to 2011. A complete list of protease (PRO) mutations, reverse transcriptase (RT) mutations, and associated drug resistance, broken down by gender and year.
(XLSX)
